# Reliability generalization meta-analysis of orthorexia nervosa using the ORTO-11/12/15/R scale in all populations and language versions

**DOI:** 10.1186/s40337-024-00997-y

**Published:** 2024-03-19

**Authors:** Leena Alshaibani, Ahmed Elmasry, Ahmed Kazerooni, Joud Alsaeed, Khalwa Alsendy, Reem Alaamer, Zainab Buhassan, Raghad Alaqaili, Hadeel Ghazzawi, Seithikurippu R. Pandi-Perumal, Khaled Trabelsi, Haitham Jahrami

**Affiliations:** 1 Department of Psychiatry, College of Medicine and Medical Sciences, Arabian Gulf University, Manama, Bahrain; 2 Department of Family and Community Medicine, School of Medicine, University of Jordan, Amman, Jordan; 3 Department of Nutrition and Food Technology, School of Agriculture, University of Jordan, Amman, Jordan; 4 Division of Research and Development, Lovely Professional University, Phagwara, Punjab 144411 India; 5 Saveetha Medical College and Hospitals, Saveetha Institute of Medical and Technical Sciences, Saveetha University, Chennai, Tamil Nadu India; 6 High Institute of Sport and Physical Education of Sfax, University of Sfax, 3000 Sfax, Tunisia; 7 Research Laboratory: Education, Motricity, Sport and Health, EM2S, LR19JS01, University of Sfax, 3000 Sfax, Tunisia; 8 Government Hospitals, Manama, Bahrain

**Keywords:** Alpha, Internal consistency, Ortho-15, Orthorexia, Psychometric, Reliability, Validity

## Abstract

**Background:**

The ORTO scale was developed in 2004 as a self-report questionnaire to assess symptoms of orthorexia nervosa (ON). ON is an unhealthy preoccupation with eating healthy food. The scale aims to measure obsessive attitudes and behaviors related to the selection, purchase, preparation, and consumption of pure, healthy food. Since its development, the ORTO-15 has been adapted into several shorter versions. The objective was to conduct a reliability generalization meta-analysis of the ORTO scale and its variant versions in all populations and languages.

**Methods:**

A systematic literature search was conducted to identify studies reporting the internal consistency of ORTO. Random-effect models were used to evaluate summary statistics of reliability coefficients, weighting the coefficients by the inverse variance using the restricted maximum likelihood method. The heterogeneity among the reliability coefficients was evaluated and assessed using numerous statistical metrics. The tau (τ), tau^2^ (τ^2^), I^2^, H2, R2, df, and the Q-statistic are among those obtained. Meta-regression analyses were used to examine moderators such as age and sex.

**Results:**

Twenty-one studies (k = 21) involving 11,167 participants (n = 11,167) were analyzed. The overall effect estimate on internal consistency was 0.59 (95% CI 0.49–0.68), with a minimum reliability coefficient of 0.23 and a maximum reliability coefficient of 0.83. The heterogeneity statistics were found to have an I^2^ of 99.31%, which suggested high heterogeneity owing to a decrease in the confidence interval (95% CI) and an increase in variability. Sensitivity analysis revealed that a few studies strongly influenced the overall estimate. Egger’s test suggested possible publication bias. Neither age nor sex significantly moderated reliability via meta-regression.

**Conclusions:**

The ORTO scale has a relatively low pooled reliability coefficient. Alternative ON assessment tools with enhanced psychometric properties are needed. Clinicians should not base diagnoses or treatment decisions on ORTO alone. Comprehensive psychiatric assessment is essential for accurate ON evaluation.

**Supplementary Information:**

The online version contains supplementary material available at 10.1186/s40337-024-00997-y.

## Abstract (plain language summary)

This review looked at the reliability of the ORTO scale and its shortened versions for assessing orthorexia nervosa (an unhealthy obsession with eating healthy foods). The researchers analyzed data from 21 previous studies involving over 11,000 participants. Results showed that the ORTO scale had relatively low reliability in consistently measuring orthorexia symptoms across studies. The summary reliability score was 0.59 on a 0 to 1 scale, with individual study scores ranging from 0.23 to 0.83. There was a significant inconsistency across the different study results. We concluded that the ORTO scale has low reliability overall for diagnosing orthorexia nervosa. New assessment tools with better measurement properties are needed. Clinicians should not rely solely on the ORTO scale, but should conduct a comprehensive psychological evaluation to properly assess for orthorexia.

**Supplementary Information:**

The online version contains supplementary material available at 10.1186/s40337-024-00997-y.

## Introduction

### Background

The term orthorexia nervosa (ON) refers to an excessive obsession with eating healthy foods and an obsessive urge to control the biological purity of the foods consumed [1, 2]. Thus, ON can lead to severe dietary restrictions [1, 2]. Initially, ON was proposed as a type of eating disorder that is similar to anorexia [1, 2]. nervosa. However, the distinction between them is based on the control of food quality rather than quantity, as well as the absence of body image disorders [3].

It must be acknowledged that ON is not yet formally classified as an eating disorder, and emerging research suggests that negative body image may contribute to the development of ON symptoms [4]. A recent study revealed that overvaluation of shape and weight specifically predicts increases in ON symptoms over time [4]. This finding indicates that certain facets of negative body image uniquely confer risk for ON [4]. However, additional research using longitudinal designs is needed to clarify which components of body image are implicated and how they interact with ON symptoms over time.

Donini et al. [5] developed the ORTO-15 scale to assess the intensity of ON behaviors. The scale was formulated based on the Bratman Orthorexia Test (BOT) and the Minnesota Multiphasic Personality Inventory (MMPI). There have been several language adaptations, including the Turkish ORTO-11 [1, 6] and the Hungarian ORTO-11-HU [7]. Stochel et al. [40] validated the ORTO-15 scale in the 15–21 year age group in Poland, and Brytek-Matera et al. [8, 9] validated it in the 18–35 year age group. The ORTO scale has also been translated into other languages, such as Arabic [10], Greek [11], German [12], and Spanish [13]. They have also been applied to clinical and nonclinical populations [14, 15].

The prevalence of ON was reported to be 74.5% among university students in Liban et al. [16], 28.3% among Polish students [17], and 49.5% among American dieticians [18]. Due to the varied prevalence and unstable factorial structure of the ORTO scale, Rogoza and Donini refined the original scale, which included the six best-fit items from the ORTO scale [19].

### Objectives

While several studies have highlighted some issues with the reliability of the ORTO scale for assessing ON, a systematic review and meta-analysis on this topic are lacking. Several individual studies have noted low internal consistency and other psychometric flaws [15, 20, 21], though some have suggested adequate [22] or high reliability [10, 13, 23, 24]. This meta-analysis aimed to obtain a more accurate overall reliability coefficient estimate and investigate the reliability coefficient among the various adaptations of the ORTO scales (all populations and language versions).

Meta-analysis serves several key functions that motivated its use in this review. First, pooling data from multiple studies increases the statistical power to detect effects that individual studies may lack sufficient power to find [25]. Second, using additional data improves the precision of effect size estimates [25]. Third, combining studies allows the examination of consistency and sources of heterogeneity, helping to resolve controversies arising from seemingly contradictory results [25]. Finally, meta-analysis can address questions not fully answered by any single study, such as the influence of language on ORTO reliability [25]. By increasing power, improving precision, clarifying inconsistencies, and answering novel questions, this meta-analysis aimed to provide enhanced evidence regarding the psychometric issues of ORTO.

## Materials and methods

This review utilized the REGEMA (REliability GEneralization Meta-Analysis) guidelines to improve the reporting quality of the meta-analysis [26]. The checklist is available as Additional file 1.

### Selection criteria

For the inclusion criteria, the review focused on studies that used the ORTO scale and its adaptations, including ORTO-R, ORTO-11, ORTO-12, and ORTO-11. The original ORTO aka ORTO-15 is a 15-item scale scored on a 4-point Likert scale, with total scores ranging from 15 to 60 [5]. Lower scores indicate higher ON risk [5]. The scale aims to measure obsessive attitudes and behaviors related to the selection, purchase, preparation, and consumption of pure, healthy food [5]. The internal consistency was the type of reliability that was investigated in this meta-analysis. The two common metrics used to assess internal consistency that were included in this meta-analysis were Cronbach's alpha [27] and McDonald's omega [28]. Cronbach's alpha is the most widely used method for evaluating internal consistency [27]. The correlation between each item and the total reliability coefficient was calculated for all the other items [27]. The values range from 0 to 1, with higher values indicating greater internal consistency [27]. McDonald's omega is considered an improvement over Cronbach's alpha, as it makes less restrictive assumptions [28]. Like alpha, omega values range from 0 to 1, with higher values indicating greater internal consistency [28]. For both metrics, values above 0.7 or 0.8 are considered acceptable in most scenarios [27, 28]. An alpha or omega greater than 0.9 generally indicates excellent internal consistency [27, 28]. Values less than 0.5 are usually unacceptable, suggesting that the items do not reliably measure the same underlying construct [27, 28].

There were no language, geographical, or cultural restrictions that affected the search for the studies [5, 7, 10, 11, 13, 19–22, 24, 29–42].

### Search strategy

The articles were identified through the following databases: Embase, PubMed/MEDLINE, and Scopus from January 2004 until June 2022. The relevant keywords used for the search were the as follows: List (1) reliability, validity, psychometric, internal consistency (Cronbach's alpha or McDonald's omega); and List (2) orthorexia and ORTO*.

The search, screening, and selection process is depicted in the REGEMA flow diagram available in the Additional file 2.

### Data extraction and quality assessment

Two authors (RA and HG) independently extracted and coded all the studies that used the ORTO scale, from which they computed the internal consistency. Disagreements between the coders were resolved by discussion with a third author (LA). No transformation methods were applied to the extracted data. To assess interrater reliability for study screening and data extraction, two reviewers independently performed each step. Cohen's kappa was used to quantify the level of agreement between reviewers at each stage [43, 44]. For the title and abstract screening stage, Cohen's kappa was 0.95 (95%), indicating excellent agreement. For full-text screening, Cohen's kappa was 0.96 (96%), also reflecting outstanding agreement. Cohen’s kappa for data extraction was 0.98 (98%) before discussion and consensus. After resolving any discrepancies through discussion, a full agreement of 100% was reached.

The methodological quality of the included studies was assessed using a modified version (COnsensus-based Standards for the selection of health status Measurement INstruments) (COSMIN checklist), which evaluates the rigor of studies on measurement properties [45]. The COSMIN was used to rate the data concurrently with the data extracted (by the same authors, RA and HG) to systematically rate each study on relevant quality criteria.

### Reported reliability, estimating reliability induction and other sources of bias

**2.5 Statistical mode, weighting method, heterogeneity assessment, and moderator analyses**

Random effects models have been used to compute summary statistics of reliability coefficients, thereby weighting the coefficients by the inverse variance [33]. The restricted maximum likelihood (REML) method was used to estimate the variance between studies. The 95% confidence intervals (95% CI) were calculated using the improved method proposed by Hartung and Knapp [33].

Heterogeneity was assessed using the τ, τ^2^, I^2^, H2, R2, df, and Q-statistic [46]. Both τ^2^ and τ are measures of the dispersion of true effect sizes between studies in terms of the scale of the effect size [47]. Moreover, τ^2^ is defined as the variance of the true effect sizes. However, τ is defined as a measure that approximates the standard deviation of true effect sizes with the presumption that these true effect sizes are normally distributed. It is useful to indicate the prediction interval. A τ^2^ = 0 suggested little or no heterogeneity, and an increasing τ^2^ indicated increasing heterogeneity [47]. The I-squared statistic (I^2^) represents the proportion of the total variance between studies that is due to heterogeneity instead of sampling errors [48]. It is expressed as a percentage with a range of 0 to 100%. It is a relative metric, so its usefulness is controversial. Values of 25%, 50%, and 75% were considered small, moderate, and large amounts of heterogeneity, respectively [49]. When I^2^ was low, there was no heterogeneity, and such analysis was not needed [49]. When I^2^ is high, a moderator or subgroup analysis could be recommended [49].

H2 was defined as the ratio of the standard deviation of the estimated overall effect size from a random-effects meta-analysis to the standard deviation from a fixed-effect meta-analysis [50]. The Q-statistic, also known as "Cochrane’s Q", is known to be a chi-squared (χ^2^) statistic and is defined as the weighted sum of squared differences between the observed effects and the weighted average effect [51]. A low p-value indicates that there is potentially some (undetermined) degree of heterogeneity [51].

The risk of publication bias was examined using the Fail-Safe N test, Egger’s test, funnel plot inspection, and Kendall's τ test, which were used to interpret the results [52]. The difference in fits (DFFITS) value was used to indicate the influence of any study after excluding that study from the model [53]. We carried out sensitivity analyses and determined several influential case diagnostic outcomes of the studies, including externally standardized residuals, Cook's distances, DFFITS values, covariance ratios, leave-one-out estimates of the amount of heterogeneity, and leave-one-out values of the test statistics for heterogeneity, hat values, and weights [54]. We determined the r-student function and discovered that all studies had externally standardized residuals between the critical values (− 1.96 and + 1.96) [54]. This is indicative of the absence of outliers in the selected studies [54].

To examine the potential moderating effects of age and sex on the overall estimate, we performed meta-regression analyses as part of our analyses [49]. We included age and sex as independent variables in the meta-regression models while using the overall estimate of reliability as the dependent variable. The meta-regression analyses allowed us to assess whether these variables significantly influenced the relationship under investigation and may also explain the heterogeneity.

### Software

R-statistical software was used to conduct the statistical analyses. version 4.3.0, which was released on 2023-04-21. A p-value less than 0.05 was considered to indicate statistical significance. The packages used were “meta” [55] and “metafor” [56].

## Results

### Results of the study selection process

Utilizing the REGEMA flowchart, a systematic review of the literature was conducted. Initial searches of the electronic databases yielded 103 records, with one additional record identified through ResearchGate, totaling 104 initial records. These records were screened based on relevance, resulting in 47 empirical studies retained for full-text assessment. Further evaluation of eligibility led to the exclusion of 12 theoretical publications, reviews, meta-analyses, and non-English articles. The remaining 35 empirical studies applied the ORTO scale (and its variants) and were deemed eligible for inclusion. However, only 21 (in twenty published studies) of these studies reported a reliability coefficient suitable for meta-analysis. The absence of the target statistic precluded the other 14 studies from quantitative synthesis. The REGEMA flow diagram is shown in Additional file 2.

The total sample in this review included n = 11,167 participants, ranging from 50 to 1289. The mean age was 27.3 years, and there was a predominance of female participants (71.5% on average). The samples came from general adult populations as well as specific groups such as university students, dietitians, vegetarians/vegans, and high school students. The studies were conducted in 12 different languages, with English (5 studies) and Spanish (4 studies) being the most common. The 15-item ORTO scale was the most frequently evaluated version (15 studies), followed by the ORTO-11, ORTO-12, ORTO-9, ORTO-7, and ORTO-R versions. The methodological quality of the studies ranged from low to high based on the COSMIN criteria. Two studies [19, 22] reported McDonald’s omega rather than Cronbach’s alpha for internal consistency. Table 1 provides a summary of the included studies. The reliability coefficients and the data are provided at https://osf.io/b8ju7.

**Table 1:** Summary of the included studies

S. no.	References	Study	Source	Language	Sample*	Scale version	Population	Sex ^(Female%)^	Mean Age ^(Years)^	Quality assessment
1	[29]	Alvarenga MS et al. 2012	Eat Weight Disord	Portuguese	392	ORTO-15	Dietitians	93%	31.7 ± 8.9	Moderate
2	[30]	Babeau C et al. 2020	Eat Weight Disord	French	768	ORTO-12	General Adults	84.8%	29.2 ± 12.9	High
3	[22]	Gkiouras K et al. 2022	Hormones (Athens)	Greek	848*	ORTO-15, ORTO-12, and ORTO-R	General Adults	66%	35.4 ± 12.1	High
4	[11]	Gonidakis F et al. 2021	Eat Weight Disord	Greek	120	ORTO-15	University Students	NR	21.6 ± 2.1	Low
5	[10]	Haddad C et al. 2020	Eat Weight Disord	Arabic	806	ORTO-15	General Adults	66.5%	27.6 ± 11.8	High
6	[34]	Heiss S et al. 2019	Appetite	English	381	ORTO-15	Vegetarian and vegans	80.8%	31.0 ± 12.9	Moderate
7	[36]	Li WL et al. 2022	Eat Weight Disord	Chinese	1289	ORTO-15 and ORTO-R	University Students	37.9%	20.9 ± 2.0	High
8	[20]	Meule A et al. 2020	Appetite	German	324	ORTO-15	General Adults	63.4%	43.4 ± 18.1	Moderate
9	[37]	Missbach B et al. 2015	PLoS One	German	1029	ORTO-15 and ORTO-9	General Adults	74.6%	31.2 ± 10.4	High
10	[38]	Mitrofanova E et al. 2021	Eat Weight Disord	English	50	ORTO-15	General Adults	60%	34.5 ± 13.8**	Low
11	[59]	Mohamed Halim Z et al. 2020	Public Health Nutr	English	286	ORTO-15	General Adults	100%	31.1 ± 11.6	Low
12	[39]	Moller S et al. 2019	Eat Weight Disord	English	585	ORTO-15, ORTO-11 and ORTO-9	General Adults	82.4%	33.4 ± 11.5**	Moderate
13	[23]	Parra-Fernandez ML et al. 2019	Eat Weight Disord	Spanish	492	ORTO-11	University Students	56.9%	19.8 ± 2.5	Moderate
14	[13]	Parra-Fernandez ML et al. 2018	PLoS One	Spanish	454	ORTO-15	University students	65%	21.5 ± 0.3	Moderate
15	[24]	Rogoza R et al. 2022	Eat Weight Disord	Arabic	363	ORTO-R	University students	61.7%	22.8 ± 3.6	Moderate
16	[19]	Rogoza R et al. 2021	Eat Weight Disord	Italian	525*	ORTO-R	General Adults	NR	NR	Moderate
17	[21]	Roncero M Study 1 et al. 2017	Span J Psychol	Spanish	807	ORTO-15 and ORTO-11	General Adults	74.1%	23.7 ± 6.4**	High
18	[21]	Roncero M Study 2 et al. 2017	Span J Psychol	Spanish	243	=	=	=	=	Low
19	[40]	Stochel M et al. 2015	Psychiatr Pol	Polish	399	ORTO-15	High School Students	63.4%	16.9 ± 1.0	Low
20	[7]	Varga M et al. 2014	BMC Psychiatry	Hungarian	810	ORTO-15 and ORTO-11	University Students	89.4%	32.4 ± 10.4	Moderate
21	[42]	Vuillier L et al. 2020	J Eat Disord	English	196	ORTO-15 and ORTO-7	General Adults	85.2%	27.9 ± NR	High

*NR* not reported

*Reported McDonald’s omega for internal consistency instead of Cronbach’s alpha, ** combined data as age reported for males and females separately, Quality assessment evaluated using the COnsensus-based Standards for the selection of health status Measurement Instruments (COSMIN)

### Pooled reliability, heterogeneity, and meta-regression

According to the random effects model, the overall effect estimate is 0.59 (95% CI 0.49–0.68), with a minimum reliability coefficient of 0.23 and a maximum reliability coefficient of 0.83; this finding suggests a low-reliability coefficient, demonstrating that the reliability and dependability of the ORTO scale are low and that there is room for statistical errors.

**Fig. 1:**
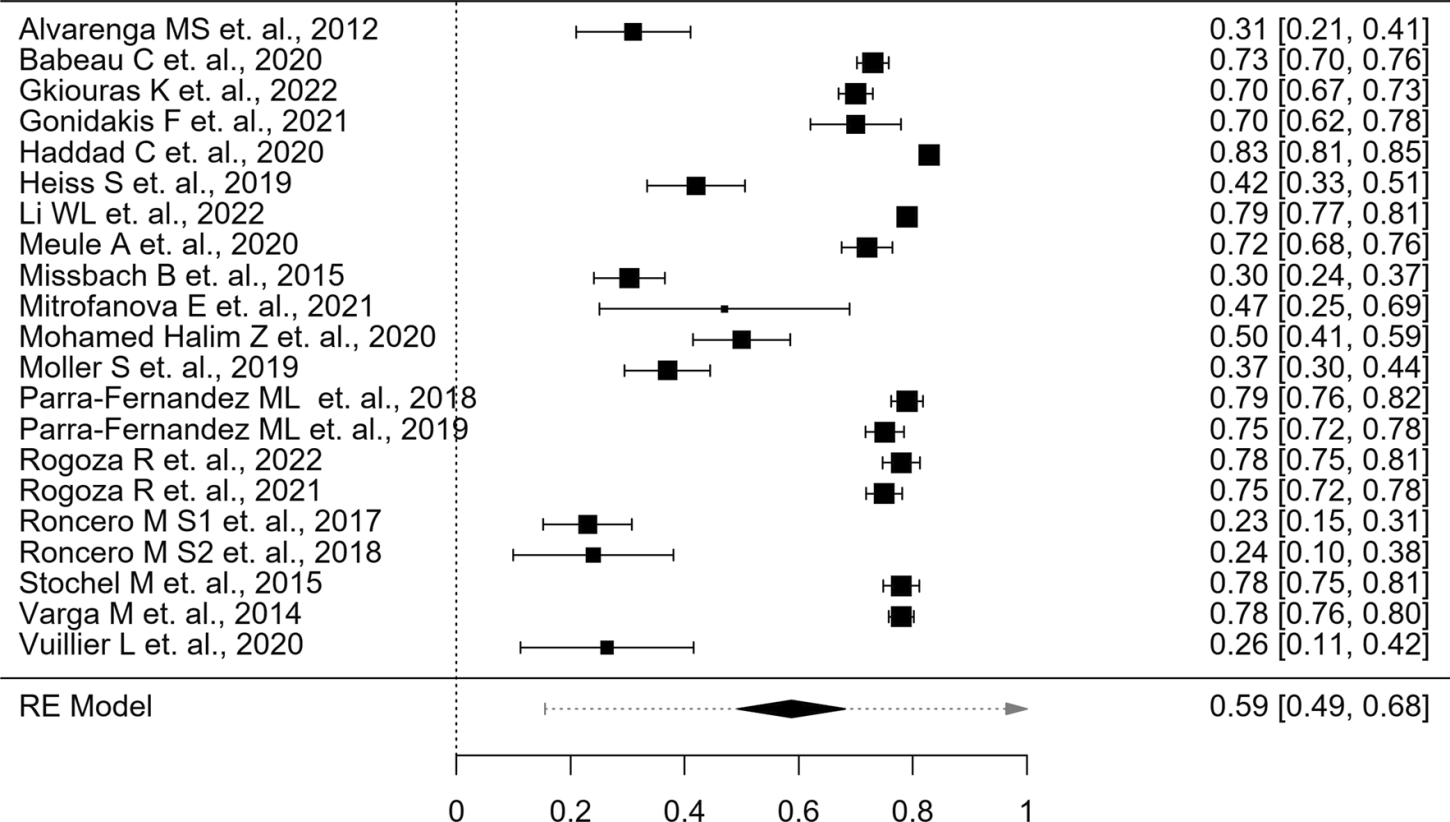
Forest plot of ORTO, without the weighing factor

The heterogeneity statistics were found to have an I^2^ of 99.31%, which suggested high heterogeneity (I^2^ > 90%) owing to a decrease in the 95% CI and an increase in variability. It is also shown that the τ^2^, or standard error, is low (SE = 0.046), estimating that p is 0.05 (0.001), explaining that the sample means are closely distributed around the population mean. Figure 1 displays a forest plot of ORTO-15 data, without consideration of the weighing factor.

**Fig. 2:**
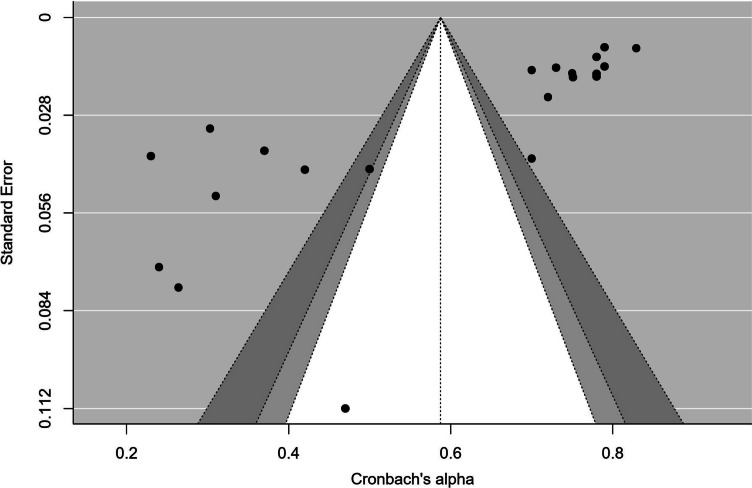
Funnel plot of Cronbach’s alpha coefficient for the dimensional ORTO scale

Publication bias was assessed by constructing a funnel plot with the follow-up statistical test Egger’s test. Egger’s test revealed a statistically significant result (p =  < 0.001). This statistically significant p-value obtained with Egger’s test indicates funnel plot asymmetry. A funnel plot of the internal consistency coefficient is shown in Fig. 2. The Fail-Safe number of Rosenthal was also determined to address publication bias (n = 21). In addition, the Kendall's τ test was performed and revealed a weak relationship (− 0.50, p < 0.001).

The results of the meta-regression analyses examining the potential moderating effects of age and sex on the overall estimate are as follows. Age did not significantly moderate the relationship (p > 0.05), suggesting that age was not a significant factor in explaining the observed heterogeneity. Similarly, the meta-regression analysis examining the moderating effect of sex indicated that sex was not a significant moderator of the overall estimate (p > 0.05). These findings suggest that neither age nor sex significantly influenced the relationship under investigation. Therefore, our results indicate that the observed heterogeneity in the overall estimate cannot be attributed to variations in age or sex across the included studies.

### Sensitivity analysis

Studies 17, 18, and 21 were found to have the lowest R scores. All the studies had Cook’s distances less than 0.15. Moreover, studies 17 and 18 had the highest Cook's distances among all the studies; i.e., these studies are the most influential. All the studies, except 17, 18, and 21, had covariance ratios higher than 1, indicating a greater influence. Cook's distance for all studies.

The study weight and overall influence of the results were also analyzed. Almost all the studies have similar weights. According to the τ^2^ results, minimal heterogeneity was noted.

## Discussion

### Summary of results

The random effects meta-analysis revealed a low overall internal consistency of 0.59 for the ORTO scale, indicating low reliability. There was high heterogeneity (I^2^ = 99.31%), implying significant variability between studies. Meta-regressions showed that neither age nor sex were significant moderators, meaning that they did not explain the heterogeneity. Although this meta-analysis investigated a large number of studies in different regions reporting reliability estimates with the data, the data obtained were only in the English language. Additionally, the meta-analysis was drawn from three databases, namely, PubMed, Embase, and Scopus, which further limited the results. Furthermore, this RG meta-analysis was based mainly on Cronbach’s alpha coefficients. Although it is familiar, commonly reported, and easy to obtain in software, it is determined to be an inappropriate measure of reliability. The alpha coefficient has been criticized as an internal consistency measure due to the inability of the τ equivalent model's restrictive assumptions to meet the test reliability coefficients [57]. Rather than using the alpha coefficient, other reliability coefficients, such as the omega coefficient, are more realistic and are always a better choice despite small samples [57].

The low reliability of the ORTO evidenced in this meta-analysis suggests its current scoring and structure might be suboptimal. Moving forward, item response theory (IRT) analysis could enhance the scale's psychometric properties [58]. IRT examines how individual items are functioning—their difficulty levels and ability to discriminate between individuals along the trait continuum [58]. This can identify problematic items for removal and support recalibrating item weighting and scoring to optimize scale reliability and validity [58]. Applying IRT methods could potentially improve the ORTO's dimensionality, reliability, and precision in assessing ON symptom severity. However, items may also need to be added or revised to better capture the underlying construct. IRT guidance coupled with a thorough expert review of item content could yield a more psychometrically sound ORTO version.

### Implications for future clinical practice

The ORTO scale has been shown to have low to questionable internal consistency reliability for use in clinical purposes, as the average alphas of the total scale and subscales were greater. On the other hand, the ORTO administration format did not affect the reliability coefficients; hence, this test could be applied online rather than face-to-face, thereby increasing its accessibility. The ORTO exhibits low to questionable internal consistency reliability; thus, the ORTO needs another measurement tool for clinical purposes to assess the ON symptomatology of people with ON disorder.

While our findings highlight significant limitations of the ORTO-15, several additional psychometric instruments have emerged for assessing orthorexic tendencies. For example, the Eating Habits Questionnaire (EHQ) [59], Düsseldorfer Orthorexie Skala (DOS) [60], and Teruel Orthorexia Scale (TOS) [61] have demonstrated acceptable internal consistency and validity [41]. Additionally, the Orion Orthorexia Nervosa Inventory (ONI) [62] was recently developed using robust scale validation methods and shows adequate reliability. Given the strong evidence for the improved psychometric properties of the ORTO-15 compared to those of the ORTO-15, we recommend that clinicians and researchers consider utilizing multiple tools for assessing ON. By employing a combination of assessment instruments, a more comprehensive and reliable understanding of ON can be obtained. This approach allows for a broader assessment of different aspects of ON and reduces the potential bias or limitations associated with relying solely on a single tool.

It must also be acknowledged that validated assessment tools can aid in the identification of orthorexic tendencies, and psychiatric evaluation remains an important component of thoroughly assessing individuals who screen positive. Scales provide an initial signal of risk but cannot be used to diagnose ON or determine specific treatment needs. Comprehensive psychiatric evaluation is essential for differentiating orthorexia from other eating or mental health disorders, given the significant symptom overlap. Expert assessment can also identify any cooccurring conditions that may warrant tailored intervention. We emphasize that screening measures should always be paired with detailed clinical interviews and examinations by an experienced psychiatrist or eating disorder specialist. Using scales as an adjunct, rather than a replacement for skilled evaluation, will enable comprehensive assessment and personalization of treatment approaches.

### Implications for future research

In research, we suggest that a second scale be used in parallel to the ORTO. There is a need for more inclusivity, which involves a wider range of variety concerning age, nationality, ethnicity, and sex, and comparisons of reliability between them. The evaluation should consider the differences between cultures and countries and how they may relate to and affect the results. Consider integrating a licensed psychiatric interview and evaluation alongside the ORTO scale to ensure more thorough and precise outcomes. Consider using another scale alongside ORTO to broaden the scope of the results.

## Conclusions

After conducting a reliability generalization meta-analysis of the ORTO scale, it was determined that the scale is weaker in measuring ON. Despite the potential of the ORTO scale to provide valuable insights into the eating habits and behaviors of individuals with ON, its lack of reliability is a significant issue. Therefore, future studies exploring ON should use alternative measures to provide more accurate and reliable data. It is important to ensure that reliable measurements are used in research studies to produce valid conclusions that can guide clinical practice and treatment options for patients.

### Supplementary Information

**Additional file 1**. REGEMA checklist.

**Additional file 2**. REGEMA flowchart.

## Data Availability

The data are available in Table 1.
