# Supplementary material for: Reliability generalization meta-analysis of orthorexia nervosa using the ORTO-11/12/15/R scale in all populations and language versions
Source: J Eat Disord. 2024 Mar 19;12:39. doi: 10.1186/s40337-024-00997-y (PMC10949742; doi:10.1186/s40337-024-00997-y)
Supplement: Supplementary file 2 — Additional file 2. REGEMA flowchart. [file 40337_2024_997_MOESM2_ESM.docx]

Empirical references excluded:

- Range of reliability coef. **(n = Nil)**

- No target reliability coef. **(n = Nil)**

Empirical references included in the meta-analysis:

**(n = 21)**

*Notes*. ^1^Systematic Reviews. ^2^Meta-analyses

Records excluded:

- Theoretical studies **(n = 9)**

- Language **(n = Nil)**

- N = 1 designs **(n = Nil)**

- SR^1^/MA^2^ **(n = 3)**

Full-text empirical references excluded:

**- No data (n = Nil)**

Records not recovered by interlibrary loan:

**(n = Nil)**

Empirical references that reported some reliability coefficient:

**(n = 21)**

Empirical references that applied the scale/s:

**(n = 35)**

Full-text empirical references assessed for eligibility:
**(n = 35)**

Empirical references screened:

**(n = 35)**

Records duplicated:

**(n= 57)**

Records screened:

**(n = 47)**

Additional records identified through other sources:

- ResearchGate **(n = 1)**

Records identified through database searching:

- Electronic databases **(n = 103)**
